# The Quality Evaluation of Rare Disease Registries—An Assessment of the Essential Features of a Disease Registry

**DOI:** 10.3390/ijerph182211968

**Published:** 2021-11-15

**Authors:** Salma Rashid Ali, Jillian Bryce, Yllka Kodra, Domenica Taruscio, Luca Persani, Syed Faisal Ahmed

**Affiliations:** 1 Developmental Endocrinology Research Group, Royal Hospital for Children, University of Glasgow, Glasgow G51 4TF, UK; salma.ali@glasgow.ac.uk; 2 Office for Rare Conditions, University of Glasgow, Glasgow G51 4TF, UK; jillian.bryce@glasgow.ac.uk; 3 National Centre for Rare Diseases, Istituto Superiore di Sanità, 00161 Rome, Italy; y.kodra@sanita.it (Y.K.); domenica.taruscio@iss.it (D.T.); 4 Division of Endocrine and Metabolic Diseases, Istituto Auxologico Italiano, 20100 Milan, Italy; luca.persani@unimi.it; 5 Department of Biotechnology and Translational Medicine, University of Milan, 20133 Milan, Italy; 6 Department of Medicine, Division of Endocrinology, Leiden University Medical Center, 2333ZA Leiden, The Netherlands

**Keywords:** registries, databases, quality, rare diseases, rare conditions

## Abstract

Rare disease (RD) registries aim to promote data collection and sharing, and facilitate multidisciplinary collaboration with the overall aim of improving patient care. Recommendations relating to the minimum standards necessary to develop and maintain high quality registries are essential to ensure high quality data and sustainability of registries. The aim of this international study was to survey RD registry leaders to ascertain the level of consensus amongst the RD community regarding the quality criteria that should be considered essential features of a disease registry. Of 35 respondents representing 40 RD registries, over 95% indicated that essential quality criteria should include establishment of a good governance system (ethics approval, registry management team, standard operating protocol and long-term sustainability plan), data quality (personnel responsible for data entry and procedures for checking data quality) and construction of an IT infrastructure complying with Findable, Accessible, Interoperable and Reusable (FAIR) principles to maintain registries of high quality, with procedures for authorized user access, erasing personal data, data breach procedures and a web interface. Of the 22 registries that performed a self-assessment, over 80% stated that their registry had a leader, project management group, steering committee, active funding stream, website, and user access policies. This survey has acceptability amongst the RD community for the self-quality evaluation of RD registries with high levels of consensus for the proposed quality criteria.

## 1. Introduction

A rare disease (RD) is defined by the European Union (EU) as a life-threatening or chronically debilitating condition with a prevalence of less than 5 per 10,000 [1]. A patient disease registry is an organized system that uses methods to collect uniform data (clinical and other) to evaluate specified outcomes for a population defined by a particular dis-ease, condition, or exposure, and that serves one or more predetermined scientific, clinical, or policy purposes [2]. RD registries aim to promote data sharing between members of the multidisciplinary team with the overall aim of improving patient care [3]. They also have a key role in supporting European Reference Networks (ERNs) for RDs [4]. In recent years, there has been a proliferation of registries for rare conditions, with over 750 RD registries currently reported to exist within Europe [5]. A concern in maintaining the dynamic of these registries is sustainability, as, not only are the infrastructures costly but they also require long-term participation by users who input the data and researchers who require data access [6]. Thus, it is imperative that recommendations relating to the minimum standards necessary to develop and maintain high-quality registries are publicized for new and existing registries. By prioritizing ethical and legal standards, high quality registries can provide access to data on a platform that ensures data security and patient confidentiality. Kodra et al. recently outlined a framework and criteria for quality management of RD registries [7]. These criteria included the establishment of a good governance system, identification of correct data sources, development of data elements and standardization, construction of a suitable IT infrastructure complying with FAIR (Findable, Accessible, Interoperable, Reusable) principles to make data available for wider use [8], production of quality data and dissemination of quality information. Moreover, this quality framework should encompass the development of adequate documentation, provision of staff training and data quality audit. Strategies that facilitate dissemination of research activities and promote wider involvement of stakeholders also ensure adaptability and sustainability of RD registries.

A recent study reported the results of a survey amongst expert centers within the European Reference Network for Rare Endocrine Conditions (Endo-ERN), a search of Orphanet [9] and RD-Connect [10] for international registries for rare endocrine conditions that exist within Europe. The study found that international registries existed for 76% of conditions covered within Endo-ERN, and experts were aware of less than half of the registries that currently exist for rare endocrine conditions [11]. Therefore, not only is there a need to improve the awareness and participation in existing registries, but it is also vital to understand whether existing registries comply with quality recommendations [7]. Moreover, an understanding of the level of consensus and the use of a standardized set of quality criteria will enable RD registries such as the European Registries for Rare Endocrine Conditions (EuRRECa; eurreca.net) to develop a pathway of vetting high quality registries with whom data can be shared [12,13].

The aim of this international study was to survey registry leaders and coordinators of RD registries to ascertain the level of consensus amongst the RD community regarding the quality criteria that should be considered essential features of a disease registry, and therefore considered as inclusion criteria to the EuRRECa platform. The survey also aimed to evaluate the extent to which existing international registries meet the proposed quality criteria and understand the extent of variation that may exist.

## 2. Materials and Methods

### 2.1. International Survey

A EuRRECa project group consisting of Work Package 3 (Quality Assurance & Evaluation), in close collaboration with Work Package 5 (Patients, Parents & Ethics), identified a small number of criteria from the quality management framework outlined by Kodra et al. [7], that could be regarded as essential for the assessment of quality of a RD registry. These criteria were incorporated into a simple online survey that could also be used for self-assessment by RD registries. The survey was performed in English using Webropol (Helsinki, Finland), a secure online platform that is endorsed and supported by NHS Greater Glasgow & Clyde and NHS Scotland. All information within Webropol (Helsinki, Finland) is kept in compliance with the UK Data Protection Act (2018) and General Data Protection Regulation (GDPR 2016/679). Registry leaders or coordinators including former participants of the International Summer School on Rare Disease Registries (*n* = 16), RD-Connect registry contacts (*n* = 296), and registry leaders representing international registries for rare endocrine conditions (*n* = 31) were invited to participate in an online survey regarding the quality criteria that should be considered essential features of a disease registry. Moreover, registry leaders from international registries for rare endocrine conditions (*n* = 31), identified in a previous mapping exercise [11], were asked to report on the extent to which their registry met the proposed quality criteria for a RD registry. The survey was performed in the last quarter of 2020.

### 2.2. Data Collection

Respondents were asked to complete contact details including name, email, institution, and the name of registry/registries that they were representing. The survey contained 17 items divided into 3 domains (Table 1). The items in the first section related to governance (e.g., ‘Registry should have a named lead’), with items in subsequent sections enquiring regarding data quality (e.g., ‘Core data elements in the registry should have a clear definition and coded values’) and IT infrastructure criteria (e.g., ‘Registry should have a web interface’) for RD registries. Respondents were asked to indicate their level of agreement with each item by selecting ‘Agree’ or ‘Disagree’ responses. If disagreeing with an item, respondents were provided with a free text option for further comment. An additional section at the end of the survey sought to obtain feedback from the respondent regarding acceptability of the length of the survey, clarity of questions, any other criteria that should be considered as essential for the quality assessment of a RD registry and any other issues that the respondent may wish to comment on regarding quality criteria for RD registries.

### 2.3. Statistical Analysis

Categorical data were analyzed using descriptive statistics. Numerical data were collated and analyzed using Minitab version 18 statistical software (Minitab LLC, State College, PA, USA).

## 3. Results

### 3.1. Survey Response

A total of 35 registry leaders representing 40 RD registries responded to the survey regarding the quality criteria that should be considered essential features of a disease registry. Of the 40 RD registries, 10 (25%), 8 (20%), and 1 (3%) were coordinated from the USA, UK, and Canada, respectively. The remaining 21 (53%) registries were coordinated from a total of seven other European countries. Of the 31 international registries for rare endocrine conditions that were identified in a previous mapping exercise [11], 22 (71%) performed the current self-assessment survey, reporting the extent to which their disease registry met the proposed quality criteria for a rare disease registry (Table 2).

### 3.2. Essential Quality Features of a Rare Disease Registry

#### 3.2.1. Governance

Regarding registry governance quality criteria that should be considered essential features of a disease registry, all registry leads agreed that a registry should have ethics approval. Of the 35 registry leads, 34 (97%) agreed that a registry should have a management team, a long-term sustainability plan, and a document outlining its standard operating protocol. A named lead and publicly accessible consent forms and participant information sheets were deemed essential by 33 (94%) respondents; 32 (91%) agreed that a registry should disseminate its activity through a report or newsletter. Of the 35 registry leads, 25 (71%) agreed that patients should be involved in the governance of a registry. Some respondents indicated that while best practice may suggest patient involvement in the governance of a registry, there are some scenarios where this may not be applicable, for example, the role of a patient in a physician driven registry may be minimal.

#### 3.2.2. Data Quality

Regarding data quality criteria that should be considered essential features of a disease registry, almost all registry leads (*n*, 34; 97%) agreed that a registry should specify who is responsible for entering the clinical data and a registry should have procedures for checking data quality. Of the 35 registry leads, 32 (91%) agreed that the core data elements in a registry should have a clear definition and coded values and 30 (86%) agreed that training should be provided to all registry users. Some respondents commented that clinical users of the registry may not require training if data input is clear and intuitive. However, researchers or other stakeholders may require formal training.

#### 3.2.3. IT Infrastructure

Regarding IT infrastructure criteria that should be considered essential features of a disease registry, all registry leads agreed that a registry should have clear procedures that only allow authorized users to have access to registry data. Of the 35 respondents, 33 (94%) agreed that a registry should have clear procedures for erasing personal data when requested; 32 (91%) agreed that a registry should have a web interface and data breach procedures in place, and 28 (80%) agreed that the web interface should allow uploading and downloading of data. Respondents commented that whilst it may be useful to have the facility to upload and download data, uploading may only be feasible and less time constraining if the same data field structures are present within databases.

### 3.3. Self-Assessment of Essential Quality Criteria for Rare Disease Registries

#### 3.3.1. Registry Governance

Of the 22 registries for international rare endocrine registries, 21 (95%) had a registry lead and project management group; 19 (86%) had a document available outlining the standard operating protocol for the registry. The majority of registries (*n*, 18 (82%) had a steering committee and an active funding stream. Moreover, 17 (77%) registries had data access policies and data sharing agreements, with 16 (73%) also specifying that they had a data access committee, patient consent forms and a registry newsletter. Around half (*n*, 12; 55%) reported involvement of patient organizations.

#### 3.3.2. Data Quality

Of the 22 registries for international rare endocrine registries, 16 (73%) reported that their registry had data element definitions, with 13 (59%) specifying the availability of personnel responsible for data entry. Around half of registries (*n*, 12; 55%) performed data quality checks, and 11 (50%) embarked on user training for clinical users of the registry.

#### 3.3.3. IT Infrastructure

Of the 22 registries for international rare endocrine registries, the majority had a registry website and authorized user access policies, as reported by 21 (95%) and 20 (91%) of registries, respectively. Data erasure procedures and data breach procedures were reported to be in place for 16 (73%) and 14 (64%) of registries. Less than half of registries had data available for upload and download (*n*, 9; 41%).

## 4. Discussion

RD registries are vital to enable research and to improve healthcare planning and delivery. The vast expansion of RD registries that has been noted over recent years necessitates the need for a simple survey that can be used to assess the quality of RD registries against recommendations outlined by expert groups and patient organizations [7]. In this paper, we report the results of an international survey of registry leaders representing 54 registries, providing objective insight into quality criteria considered essential for RD registries and the results of self-assessment, including aspects of governance, data quality and IT infrastructure.

There was a high level of consensus amongst the respondents on a large majority of quality criteria that should be considered as essential features of a RD registry. Regarding registry governance, all respondents agreed that ethics approval should be mandatory, with almost all indicating that a registry management team and long-term sustainability plan would be preferable for a high-quality registry. Ensuring sustainability through clear policies that are acceptable to patients, health care providers, researchers and industry for data provision and data access coupled with widespread dissemination and knowledge exchange through closely affiliated professional societies and patient support groups is vital. Interestingly, approximately 30% of respondents indicated that patient involvement would not be an essential criterion. However, the involvement of patients and patient organizations may be advantageous, with previous studies showing that patient involvement complements the research emphasis of registries, and most RD patient organizations have goals to promote or support research of their condition [14,15].

Regarding data quality criteria, almost all respondents (97%) agreed that personnel responsible for data entry and procedures for checking data quality should be specified, with the majority (91%) also agreeing that core data elements should have clear definitions and coded values. Opinions regarding data quality and governance appear to be well-aligned across the RD community and other stakeholders including industry [16]. High quality data is an important element in the maintenance of a registry and data quality can be assessed via a number of dimensions including: data completeness, validity, coherence and comparability, accessibility, usefulness, timeliness, and prevention of duplicate entries [17,18,19]. The European Platform on Rare Diseases Registration (EU RD Platform) developed via the European Commission through its Directorates-General Joint Research Centre (DG JRC) and Health and Food Safety (DG SANTE) also aims to set European-level standards for data collection and data sharing, enabling interoperability and sustainability for existing RD registries in Europe, facilitating the production of high quality data from these registries [20]. Regarding the IT infrastructure of a high-quality registry, all respondents stated that registries should have clear procedures for allowing only authorized user access to data, with the majority also specifying that registries should have clear procedures for erasing personal data and data breach procedures in place.

Our survey showed that there does appear to be some variation in the governance of existing endocrine registries within Europe. Nevertheless, over 80% of registries that performed the self-assessment using the survey tool stated that their registry had leadership, a project management group, a steering committee, an active funding stream, a web interface and user access policies. More than 70% of registries also reported to have data element definitions within their platform.

The strengths of this exercise were that an international perspective was obtained regarding levels of agreement for the quality criteria considered essential for an RD registry, with responses from registry leaders representing over 50 RD registries across a range of medical specialties. All respondents stated that the overall length of the survey was acceptable. Obtaining more detailed information through provision of further quality selection criteria would have perhaps been advantageous, however, a balance needed to be struck between maximizing the information available for collection and reducing respondent burden. It must also be acknowledged that whilst the criteria outlined in this survey may be considered essential quality criteria for a RD registry, fulfilling these recommendations may be challenging in resource limited settings where funding is restricted. Of the 353 registries that were approached to participate in the current survey, 54 responded. It is possible that those registries that had a greater level of adherence to the proposed quality standards responded to the survey. In addition, several former participants of the annual International Summer School on Rare Disease Registries were amongst the survey respondents. This event plays an important role in the education and training of those involved in RD registries and forms part of a series of training activities that have been proposed by the European Joint Programme on Rare Diseases (EJP RD). Going forward, there is a need for such training courses to engage with a greater number of RD registries from a wider range of geographical and resource settings.

Of the 272 registries surveyed by the European Platform For Rare Disease Registries (EPIRARE), a European Union (EU)-funded project (‘Building Consensus and Synergies for the EU Registration of Rare Disease Patients’), 48% did not have a clear strategy for long-term sustainability, 34% did not have a specific management group, 30% did not share data, and 21% were established without any clear funding [21]. In spite of the heterogeneity of the European registries, a survey performed by EPIRARE amongst European RD registries identified the following requisites for registries: financial support, motivation of data providers, data quality assessment, improvement of communication and visibility and extension of collaborations. Moreover, the registry holders were supportive of a common EU platform for RD registries [21,22].

## 5. Conclusions

The simple online quality assessment demonstrates acceptability amongst the RD community. It may be used for the quality evaluation of RD registries and enables assessment and improvement of organizational aspects of RD registries to ensure their sustainability. A survey like this could be used by networks to develop objective criteria that allows them to collaborate and engage with registries of an optimal quality.

## Figures and Tables

**Table 1:** Questionnaire items.

Survey Domain	Item	Response Type
Contact details for respondent	Name Email Institution Registry/Registries ^a^	Free text
Governance	The registry should have a named lead The registry should have a management team Patients should be involved in the governance of the registry The registry should have a long-term sustainability plan The registry should have ethics approval The registry should have publicly accessible consent forms and participant information sheets The registry should have a document outlining its standard operating protocol The registry should disseminate its activity through a report or a newsletter If you disagree with any of the above criteria, please comment:	Select one option for each item (Agree or Disagree) Free text
Data quality	The core data elements in the registry should have a clear definition and coded values The registry should specify who is responsible for entering the clinical data The registry should have procedures for checking data quality The registry should provide training to all users If you disagree with any of the above criteria, please comment:	Select one option for each item (Agree or Disagree) Free text
IT infrastructure	The registry should have a web interface The web-interface should allow uploading and downloading of data The registry should have data breach procedures in place The registry should have clear procedures for erasing personal data when requested The registry should have clear procedures that only allow authorized users to have access to registry data If you disagree with any of the above criteria, please comment:	Select one option for each item (Agree or Disagree) Free text
Feedback	Was the length of the survey acceptable? (Please specify time taken for completion) Could any of the questions be clearer? Are there other criteria that should be considered as essential? Are there any other issues that you would like to comment on?	Select one option (Yes or No) Please specify (free text)

^a^ Mandatory field.

**Table 2:** Rare disease registries represented by survey respondents.

Registries Reporting on Essential Quality Features of a Rare Disease Registry, *n* = 40
3q29 Registry
Amyotrophic Lateral Sclerosis (ALS) Registry
Barth Syndrome Registry
Behcet Disease Registry
Canadian Neuromuscular Disease Registry
Clinical Registry investigating Bardet-Biedl Syndrome (CRIBBS)
Congenital Muscular Disease International Registry
Cystinuria: Rare Kidney Stone Consortium
EU Rare Diseases Registry for Wolfram Syndrome, Alstrom Syndrome, Bardet-Biedl Syndrome and other rare diabetes syndromes (EURO-WABB) *
European Alport Registry
European Consortium of Lipodystrophies (EcLip) *
European Network and Registry for Homocystinurias and Methylation Defects (E-HOD)
European Registry and Network for Intoxication Type Metabolic Diseases (E-IMD)
European Registry for Children on Renal Replacement Therapy (ESPN/ERA-EDTA Registry) *
European Registry for Rare Bone and Mineral Conditions (ERN BOND: EuRR-Bone)
European Registry on Cushing’s Syndrome (ERCUSYN) *
FAP Registry (Belgium)
Friedreich’s Ataxia Registry
GLUT1 deficiency
Inherited Retinal Dystrophies
International Cholangiocarcinoma Patient Registry
International Disorders of Congenital Adrenal Hyperplasia (I-CAH) Registry *
International Disorders of Sex Development (I-DSD) Registry *
International Working Group on Neurotransmitter Related Disorders (iNTD)
LGMD2A/R1 Global Registry
Leige Acromegaly (LAS) Database *
Mitochondrial Registry
Myotubular and Centronuclear Myopathy Patient Registry (MTM and CNM)
National Alpha-1 Antitrypsin Deficiency Registry
Nordinet International Outcome Study *
Poland Syndrome Registry
RenalTube Registry
Ring14 Syndrome Registry
Sarcoidosis Advanced Registry for Cures (FSR-SARC)
Spinal Muscular Atrophy (CSMA) Registry
Spinal Muscular Atrophy (SMA) Global Registry
UK Duchenne Muscular Dystrophy (DMD) Registry
Unified Registry for Inherited Metabolic Disorders (U-IMD)
X-linked Hypophosphataemia (XLH) Registry
**Registries Undertaking Self-Assessment of Essential Quality Criteria for Rare Disease Registries, *n* = 22**
ACROSTUDY (International Somavert Database)
Congenital Hypothyroidism Variant Database (UK10K_RARE_THYROID)
Cooperative European Paediatric Renal Transplant Initiative registry
COST Action BM1105 Patient Registry—GnRH Network
European LeukoDatabase (LeukoDB)
European Network for the Study of Adrenal Tumours (ENSAT)
European Neuroendocrine Tumour Society (ENETS)
International network for paediatric diabetes centers (SWEET)
International Patient Registry and Cohort for Congenital Disorders of Glycosylation (EUROGLYCANET)
National and European cohort on Imprinting Disorders and their Metabolic Consequences (RaDiCo-IDMet)
Pfizer International Growth Database (KIGS)
Pfizer International Metabolic Database (KIMS)
X-linked Adrenoleukodystrophy Database (X-ALD)
X-linked Hypophosphataemia (XLH) Registry

* Registries also involved in self-assessment of essential quality criteria.

## Data Availability

The data that support the findings of this study are available from the corresponding author upon reasonable request.
